# Exploring Myelin Dynamics in Demyelinating Disorders at the Molecular Level

**DOI:** 10.3390/cimb46030114

**Published:** 2024-02-26

**Authors:** Paschalis Theotokis

**Affiliations:** 1 Laboratory of Experimental Neurology and Neuroimmunology, 2nd Department of Neurology, AHEPA University Hospital, 54636 Thessaloniki, Greece; ptheotokis@auth.gr; 2 Laboratory of Histology and Embryology, Medical School, Aristotle University of Thessaloniki, 54124 Thessaloniki, Greece

Investigating the subtle molecular mechanisms underlying demyelinating disorders of the central nervous system (CNS) is pivotal in advancing therapeutic strategies and improving patient outcomes. This Editorial summarizes the dynamic landscape of myelin biology and demyelination, around three key themes extracted from a selection of ten seminal papers. Beginning with myelin origin and oligodendrocyte dynamics, the discussion progresses to inflammation, encompassing multiple sclerosis (MS) and the paramount role of microglia, before exploring systemic–therapeutic approaches. Through this structured approach, the aim is to unravel the molecular nuances of demyelinating disorders, offering insights for novel therapeutic interventions.

Primordial components of the myelin sheath at the embryological level are fundamental for illuminating the mechanisms driving myelinogenesis in adult life, particularly under both normal conditions and scenarios requiring repair [1]. Dermitzakis et al. explore the role of early life developmental cues and molecular drivers in myelinogenesis. The key contributors to this process include oligodendrocyte lineage cells, extensively investigated in animal models such as zebrafish [2] and mammalian CNS [3,4]. Whilst there is a myriad of molecular cues involved, Fahim et al. have identified OLIG2 and MYT1L transcription factors as essential catalysts for enhancing the differentiation potential of human mesenchymal stem cells into oligodendrocytes, thus offering promising avenues in advancing therapeutic strategies for demyelinating disorders.

Among the demyelinating disorders, the most prevalent one is MS, affecting 2.8 million people worldwide [5]. Experimental autoimmune encephalomyelitis (EAE), an animal model of MS, recapitulates the immunological aspects of the disease [6], serving as a substrate for cell therapy [7] and drug testing. With regards to the latter, Haghmorad et al. investigate the oral administration of myelin oligodendrocyte glycoprotein as an immunomodulatory agent, revealing its ability to induce Th2/Treg cells while suppressing Th1/Th17 immune responses, thereby offering an elegant strategy to attenuate EAE. Building upon this, Papiri et al. comprehensively explore the inflammatory and neuroglial aspects of MS, shedding light on the complex molecular interactions driving disease progression. Further delving into the specific manifestations of MS, Ciapă et al. meticulously examine the molecular mechanisms underlying optic neuritis, enhancing our understanding of the challenges posed by optic nerve involvement in MS.

Transitioning to the role of glial cells in inflammatory-based CNS demyelination, Dermitzakis et al. present a historical account of microglia origins, unraveling its eccentric journey within the CNS and contributing to a holistic perspective on neuroinflammation. Microglial fluctuations are intricately linked to key neurodevelopmental hallmarks and play a crucial role in regulating CNS myelin growth and integrity [8,9,10]. Microglia play a tremendous role in the healthy brain [11,12], MS-related diseased conditions [13,14] and aged CNS [15]. Notably, Piper et al. investigates the pro-inflammatory and pro-apoptotic effects of l-azetidine-2-carboxylic acid in BV2 microglial cells, offering valuable insights into potential neuroprotective strategies amidst microglial responses.

The focus shifts to diagnostic markers and therapeutic approaches addressing immune-mediated demyelinating disorders. Tonev et al. underscore the impact of plasma exchange in MS on circulatory factors, nerve growth factor and sphingosine-1-phosphate plasma levels, shedding light on the delicate balance between pathogenic factors and therapeutic interventions. Transitioning to foundational diagnostics, Kelbich et al.’s analysis of cerebrospinal fluid serves as a framework for understanding CNS impairment, laying the groundwork for unveiling molecular markers in demyelinating disorders. Lastly, Kaffe et al. scrutinize the roles of caloric restriction mimetics in central nervous system demyelination and myelin regeneration, uncovering novel therapeutic avenues to induce remyelination, a topic that has garnered significant attention in the research field [16].

In conclusion, this Editorial navigates through the complex landscape of molecular mechanisms which underlie demyelinating disorders, delineating key findings from multiple research teams. These studies offer foundational insights into oligodendrocyte dynamics and myelin origin to the elucidation of inflammation’s role in MS and the contribution of glial cells, particularly microglia. The corpus of presented papers sheds light on the multifaceted nature of demyelination. Collectively, these studies advance current knowledge, provide directions for systemic and therapeutic interventions, thus, paving the way for enhanced diagnosis, management and potential treatment options for demyelinating disorders of the CNS.
